# Medical and Physical Intelligence

**Published:** 1822-03

**Authors:** 


### Medical and Physical Intelligence.

### 1. *Treatment of Mucous Polypi.*

DR. J. B. RAINER recommends the external application of tinctura opii crocata to the mucous polypi of the nose, as an effective means for their removal. He directs the patient to rub the polypus, three or four times a-day, with the above-mentioned preparation; and states that, in three days, a very sensible diminution takes place in the polypus, which disappears altogether in the space of three weeks. Two cases of polypi, one of them situated in the ear, effectually removed by this treatment, by Dr. ANDREW, physician at Babenhausen, are related in tbe 56th Number of the Medizinischen Annalen for 1821.—*Med. Chirurg. Zeitung.*

### 2. *Tetanus successfully treated.*

Dr. RAINER has related a case of tetanus, brought on by a splinter of wood penetrating a recent wound. The means employed on this occasion were copious blood-letting, anodynes, and frictions with mercurial ointment. The alarming symptoms gradually subsided, and the disease was completely subdued, although the splinter could not be extracted, and did not come out until four months after the accident. —*Med. Chirurg. Zeitung.*

### 3. *Hydrophobia.*

Considering the awful fatality attending this disease, it is not to be wondered at, that so many new remedies should be recommended for it. In the class of preservatives, we have now to record the *scarafaggio,* (scarabæus melalontha of Linnæus,) proposed by Dr. PERSON. The knowledge of the preservative properties of this insect against hydrophobia, is quite accidental, and founded on the solitary fact of a young dog, that had been bitten by another of its species known to be mad, betraying no symptoms of disease at any subsequent period, after having casually eaten a large quantity of the above-mentioned insect.—*Mediz. Chirurg. Zeitung.*

### 4. *Population of Great Britain.*

The following is a comparative statement of the population of the several counties of Great Britain, in the years 1801, 1811, and 1821:

| | Population 1801. | Rate of Increase per Cent. | Population 1811. | Rate of Increase per Cent. | Population 1821. |
|---|---|---|---|---|---|
| ENGLAND ...... | 8,331,434 | 14½ | 9,538,827 | 18 | 11,260,555 |
| WALES ........ | 551,546 | 13 | 611,788 | 17 1-5 | 717,108 |
| SCOTLAND ..... | 1,599,068 | 13 | 1,805,688 | 15 6-7 | 2,092,014 |
| | 10,472,048 | 14 | 11,956,303 | 17 2-3 | 14,069,677 |
| ARMY, NAVY, &c. | 470,598 | .... | 640,500 | .... | 310,000 |
| Totals ...... | 10,942,646 | .... | 12,596,803 | .... | 14,379,677 |

*Remarks.*—1. The population of the Islands in the British Seas, not having been ascertained in 1801 and 1811, no comparative statement thereof can be given; the existing population of those Islands, when enumerated in the year 1821, appears to have been as follows, viz.—In the Isle of Man, 40,084; in the Island of Guernsey (and its dependent Islets), 20,827; in the Island of Jersey, 28,600; in the Scilly Isles, 2,614:—In all these Islands, 92,122 inhabitants.

2. The number of males ascribed to the army, navy, &c. in May 1821, is the nearest estimate that can at present be made of the regular army, the artillery, the navy, and seamen employed in registered merchant vessels. None of these classes can be ascribed to particular counties, nor consequently taken into account in the foregoing comparative statement, otherwise than in the general total; nor properly in that, without making allowance for the large number of foreigners (perhaps 100,000) employed as merchant seamen during the war, and consequently taken into account in 1811; nor without considering that many soldiers and sailors are attributable to Ireland; which consideration would operate proportionally upon the larger number of men serving in 1811, as compared with the smaller number serving in 1821. In order, therefore, to avoid the uncertainty hereby indicated, the rate of increase has been separately calculated on the respective numbers of females only, viz.—

| | 1801. | Increase per cent. | 1811. | Increase per cent. | 1821. |
|---|---|---|---|---|---|
| FEMALES... | 5,492,354 | 14 or 14.02 | 6,262,716 | 15 4-5ths or 15.82 | 7,253,728 |

And the absolute increase of population in Great Britain (if measured by that of females only) appears to have been about one million and a half in the first period, two millions in the second period.—*Parl. Pap.*

### 5. *New French Medical Journal.*

Dr. Broussais, whom we have had frequent occasions of introducing to our readers, has transmitted to us the prospectus of a new Medical Journal, entitled "Annals of Physiological Medicine;" to be edited by Dr. Broussais himself, and destined to record the principal facts and doctrines connected with what he has denominated the physio-pathological school. This journal will be published monthly, and the first number of it was to have appeared in January; but as yet we have not received it. The first part is intended to contain original communications relative to cases of disease treated according to the Editor's own peculiar system; and the analysis of medical works deserving of consideration. The second part will be entirely consecrated to the development of the Broussaiean doctrine; beginning with physiology, and gradually extending to every branch of medical studies, so arranged that, at the end of a certain period, the reader will be able to have the different parts of the journal bound separately, and will thus possess complete treatises on those various subjects.

### 6. *Population of the Russian Empire.*

According to the statement just published by the Synod (which, however, includes only the Greek Church,) the number of the births, &c. in the Russian Empire, in the year 1819, was as follows:—

| | | |
|---|---|---|
| BIRTHS.—Males | . . . . . . | 796,426 |
| Females | . . . . . . | 725,708 |
| Total | . . . | 1,522,134 |

Being 90,686 more than in the year 1818.

| | | |
|---|---|---|
| DEATHS.—Males | . . . . . . | 467,668 |
| Females | . . . . . . | 451,441 |
| Total | . . . | 919,109 |

Being 44,102 more than in the year 1818.

The number of births exceeds that of the deaths by 603,025. Among the deaths were 233,697 males under five years of age. It is worthy of remark, that, if we except the first ten years of infancy, the greatest mortality takes place at the age of 60 to 65 years, for in these years there died 17,745 males,—that is, the 26th or 27th part of the whole.

Among the deaths of the male sex, (the age of the females is not stated,) there were 18,741 above 80; 5,754 above 90; 1,094 above 100; 324 above 105; 179 above 110; 90 above 115; 56 above 120; 23 above 125; 13 above 130: and two the extraordinary age of between 140 and 150.

### 7, Dr. THOMSON, *professor of Chemistry at Glasgow, and* Mr. ALLAN, *the author of Mineralogical Tables.*

In consequence of certain unfavourable reports which Dr. Thomson had circulated against Mr. Allen of Edinburgh, an action for damages was brought by the latter against the professor.

This cause, which has excited considerable interest, and has been the subject of much conversation in the scientific circles for some time past, was appointed for trial January 25th, in consequence of which the Court was completely filled on its opening.

The grounds of action alledged by the pursuer, Thomas Allan, esq. banker in Edinburgh, were, that the defender, Dr. Thomas Thomson, Professor of Chemistry in the University of Glasgow, had expressed himself to persons in that city and its vicinity, in terms which were false and injurious to the pursuer, such as that a friend of his was about to prosecute the pursuer for appropriating to himself certain minerals, and that he had imposed upon mineral dealers. The damages were laid at 5000l.

Many applications were made to Mr. Allan, by the friends and the counsel of Dr. Thomson, to accept an apology rather than carry the question into Court; Mr. Allan uniformly declared, that being actuated by no vindictive motives to Dr. Thomson, he would not object to receive such an apology as his friend, Mr. Ferguson of Raith, approved of. It was not, however, till the evening before the trial, that the final arrangements were made, when Mr. Ferguson positively refused to listen to any thing but an apology made in open Court, and on the express condition that Dr. Thomson defrayed all the expences incurred. In consequence, when the cause was called, Mr. Cuninghame stated to the Court, that the pursuer, Mr. Allen, had accepted of an apology from the defender, and that in return Mr. Allan had agreed to give the explanation which Mr. Ferguson had drawn out.

The learned gentleman then read the following apology by Dr. Thomson.

"Having been led, by very false information, to accuse most unjustly Thomas Allan, esq. banker in Edinburgh, on a subject connected with his mineralogical pursuits, I now publicly express my sincere regret for having propagated a most groundless calumny against that gentleman; and do declare, that I now find, that so far from what was reported to me, and repeated by me, having the slightest foundation, Mr. Allan, on the contrary, was the direct means of tracing, and transmitting to the proper owner in London, the minerals which were the subject of the charge. I trust that Mr. Allan and his friends will accept of this apology for the error I have been led into.—Edinburgh, Jan. 21, 1822."

The following explanation was then read by Mr. Cockburn:

"I accept of the apology Dr. Thomson has now made, and take the opportunity which he has now afforded me, of expressing my regret for the harsh language I used towards him, which arose altogether from his repetition of injurious allegations with respect to my conduct and character."

Mr. Jeffrey said, he had only to regret that this accommodation had not been entered into at an earlier stage, but had been delayed till the latest moment that such a measure was practicable; he added, that as the delay was to be imputed to Dr. Thomson, it was understood that gentleman should defray the whole expense incurred.

This the counsel for Dr. Thomson acquiesced in, it being stated by the Court, that it was a matter of private arrangement among the parties.

The Lord Chief Commissioner felt much gratified that this Court should be instrumental in bringing about an amicable adjustment in any case, but particularly in such a case as this, where the parties were both of highly respectable character, and eminent for their scientific knowledge.

### 8. *Lithographic Botany.*

An experiment has lately been made to take off impressions from plants by lithographic printing. A specimen of *Sibthorpia Europœa*, which was gathered several years ago in Cornwall, was covered with lithographic ink and impressed on a stone, from which stone several impressions were afterwards taken. The experiment was not so successful as was wished, but still promised to be beneficial in leading to the means of multiplying copies of the impressions of plants, much more accurate, in some respects, than a drawing can be expected to be.

### 9. *Preservation of Milk.*

The following method is recommended for the preservation of milk, either at sea or in warm climates. Provide pint or quart bottles, which must be perfectly clean, sweet, and dry; draw the milk from the cow into the bottles, and as they are filled, immediately cork them well up, and fasten the corks with packthread or wire. Then spread a little straw on the bottom of a boiler, on which place the bottles, with straw between them, until the boiler contains a sufficient quantity. Fill it up with cold water; heat the water, and, as soon as it begins to boil, draw the fire, and let the whole cool gradually. When quite cold, take out the bottles, and pack them with straw or saw-dust in hampers, and stow them in the coolest part of the ship, or in a cool place. Some years since, there was a Swedish or Danish vessel at Liverpool, having milk on-board preserved in this manner; it had been carried twice to the West Indies and back to Denmark, and had been above eighteen months in the bottles; nevertheless, it was as sweet as when first milked from the cow.

### 10. *Tests for Arsenic.*

Dr. Porter, of the University of South Carolina, in observing on the tests for the detection of arsenic, remarks, that an appearance, similar to Scheele's green, is produced by carbonate of potash, added to a solution of copper containing coffee, but without arsenic, more striking than if a weak solution of arsenic be used. He also states that, in the production of Scheele's green by arsenic, sulphate of copper and carbonate of potash, chromate of potash might be substituted for the arsenic; and that the precipitate produced could not be distinguished by the eye from Scheele's green. Also that Mr. Hume's test of the nitrate of silver (as modified in its application by Dr. Marcet,) gave, with chromate of potash, a yellow precipitate, which, when placed side by side with one produced by arsenic, could not be distinguished by colour or appearance from it.—Silliman's *Jour.* iii. p. 355.

### 11. *Effects of Lightning.*

A house at Geneva, which had plates of tinned iron disposed in various ways about the roof and other parts, was struck by lightning on the 3d of July. The lightning produced various effects here and there, but among them none was more remarkable than that produced on one of the plates of tin that had been placed on the roof against the chimney. This plate is pierced with two circular holes, about an inch in diameter each, and four inches apart. Each hole is strongly burred, but the remarkable circumstance is, that the burs of the two holes are in opposite directions, which, according to M. Pictet, either indicates that the electric fluid has passed through the plate forming one hole, has moved five inches along it, and then gone through again; or that there were two currents of fluid which moved simultaneously in opposite directions, at five inches distance from each other.

### 12. *Remarkable Operation.*

Professor Gräfe, of Berlin, has removed one half of the inferior maxilla from its articulation; in consequence of which operation it became necessary to apply a ligature to the left carotid artery. The patient was a girl twenty-three years of age, who had had a bony tumor situated on the jaw ever since she was six years old, and had then grown to an enormous size. The patient supported the operation with great fortitude. The wound was dressed on the fifth day, when the patient was not only able to utter some words, but to take food without great difficulty.

16. *Internal Hemorrhage, without any apparent Rupture of Blood Vessels.*

A very rare instance of sudden death from internal hemorrhage in the abdomen, without any visible rupture of a blood vessel is related in one of the late numbers of Hufeland's Journal for 1821. A woman, aged thirty-three years, who had always regularly menstruated, had her menses suppressed, in consequence of catching cold and getting her feet wet. She fell ill the same evening, and died at noon, on the following day. On examination, no traces of inflammation could be discovered in any part of the body; but, in the abdomen, upwards of three pints of fluid blood were found without any evident cause being traced for such an occurrence. It is needless to state that the accident was attributed to the sudden cessation of the menses.—*(Hufeland's Journal.)*

17. *Indication of the Temperature of a Room by two Thermometers at different Altitudes.*

Mr. John Murray has been induced from some accidental observations to make a series of experiments on the difference of temperature indicated by two thermometers at different altitudes, though, otherwise, under similar circumstances; and was surprised to find that there existed an invariable difference between the temperature indicater by each, which he is inclined to consider to be an accurate guide for judging of the state of the weather. He placed one of the thermometers, with its ball, on the floor, and the other he suspended 6½ feet above the former. When the difference between the two exceeded 2°, to 2° 5′, F. The weather was variable and wet.—*(Phil. Mag.)*

18. *Lampyris Italica.*

M. Grotthus being lately at Rome, paid particular attention to the phosphorescent organ of the *Lampyris Italica.* This insect, plunged into water, continued luminous for several hours; under oil of olives, the light diminished after a quarter of an hour, and disappeared entirely after twenty minutes. The case was nearly the same with hydrogen gas and carbonic acid. When the insect was withdrawn from this gas and transposed immediately into an ordinary atmosphere, the phosphorescence recommenced on the instant. Some Lampyræ, in which the phosphorescent power was so far extinct that oxygen gas could not revive it, recovered it when plunged into an atmosphere of nitric vapours. When the phosphorescence became extinguished by the nitric vapour, it could no longer be developed by any other agent.—*Grotthus's Forschngen,* 1820.

19. *Atmospheric Phenomena.*

On the 25th of December last, about seven o'clock in the evening, at Hamberg, the sky being clear and serene, there was observed in the neighbourhood of Battenheim and Altendorf an igneous meteor, of a globular form, about the apparent size of a full moon, which, after taking a direction from north-east to south-east, fell to the ground and disappeared, with an explosion as loud as the report of a cannon. Its light was as strong as that of a bright flash of lightning. On the 25th the mercury in the barometer fell lower than had ever been seen by the oldest inhabitants.

This phenomenon, says a letter from Frankfort of the 31st Dec. appears to have been seen at places very distant from each other. On the nights of the 24th and 25th the mercury likewise fell at Frankfort to twenty-six inches six lines, without being accompanied by any other change in the atmosphere but a strong wind, which did not rise to a tempest as in other places. The wind was stronger in the night of the 29th, though the mercury had risen a little.—*(Phil. Mag.)*

### 20. *More Information respecting Iodine.*

M. Heusmans read before the Society of Medicine of Louvain, at its sitting of the 16th of January, 1821, a paper upon the preparation of the tincture of iodine, and the re-establishment of that tincture deteriorated by time; as also on the non-existence of iodine in burnt sponge and in the ashes of the turf of Holland and the Netherlands. MM. Fyfe and Straub had announced the presence of this comburant in the ashes of Swiss turf.

As to the means of re-establishing the tincture of iodine, when by the decomposition of the alcohol it has passed into the acid state, it consists in infusing the tincture with an excess of super-oxide of manganese; the hydrogen is expelled, and the iodine regenerated; but the alcohol does not recover its primitive force, and is thus prevented from holding the iodine in solution. This tincture, which M. Heusmans exhibited to the Society, was of a deep brownish red, it stained the hands intensely, and contained forty-eight grains of iodine per ounce of alcohol, at 35° B.—*Annales Generales des Sciences Physiques.*

*List of the Medical Officers in His Majesty's Navy, who subscribed for the Piece of Plate, value one hundred Guineas, presented to* Sir Gilbert Blane, Bart.

*Physicians to Royal Naval Hospitals and to the Fleet.*

James Magennis, M.D. Leonard Gillespie, M.D. Isaac Wilson, M.D. and J. D. H. Dickson, M.D.

*Surgeons.*

A. Anderson, Esq. Brompton
R. Atcheson, Esq. Mediterranean
R. W. Bampfield, Esq. London
R. H. Beaumont, Esq. Gravesend
T. Bishop, Esq. London
W. Burnett, M.D. Greenwich Hospital
P. Cunningham, Esq. on Service
J. Cullurne, Esq. Lisbon
R. Dobson, M.D. Chatham Marines
A. Douglas, M.D. Hospital Ship
R. Dunn, M.D. Woolwich Dock-yard
T. Elliott, Esq. London
J. Gillies, M.D. Bath
S. Godfrey, Esq. Windsor
A. B. Granville, M.D London
R. Griffiths, Esq. Mediterranean
A. C. Hutchison, Esq. London
E. G. Jones, M.D. London
J. Johnson, M.D. London
W. Jamieson, Esq. Fraserburgh
R. Kent, M.D. London

A. Leslie, Esq. Newton Abbot
R. Malcolm, Esq. Mediterranean
A. Menzies, Esq. London
J. M'Leod, M.D. late of the Alceste
H. Monk, Esq. Guernsey
G. Magrath, M.D. Plymouth
R. Newberry, Esq. Upchurch
B. F. Outram, M.D. London
D. Quarrier, M.D. Marine Artillery
S. Revans, Esq. Halesworth
G. Roddam, M.D. Royal Yacht
W. Stenhouse, Esq. Dunfermline
R. Tainsh, M.D. Woolwich Marines.

☞ See the London Medical and Physical Journal for February, where an error of the press crept into the date of Sir Gilbert's answer to the deputation. Instead of the 17th, it should have been the 27th of December.

### METEOROLOGICAL JOURNAL.

By Messrs. WILLIAM HARRIS and Co. 50, Holborn, London.

*From January 20, 1822, to February 19, 1822.*

| | MOON. | Rain gauge | THERM. | | | BAROM. | | DE LUC'S HYG. | | WIND. | | ATMOSPHERIC VARIATION. | | |
|---|---|---|---|---|---|---|---|---|---|---|---|---|---|---|
| | | | 9 A.M. | MAX. | MIN. | 9 A.M. | 10P.M. | 9 A.M. | 10P.M. | 9 A.M. | 10P.M. | 9 A.M. | 2 P.M. | 10P.M |
| Jan. 20 | | | 46 | 51 | 40 | 30.17 | 30.26 | 63 | 60 | W | WNW | Cloudy | | Fine |
| 21 | | | 42 | 48 | 39 | 30.33 | 30.37 | 62 | 62 | WNW | W | Fine | | Cloud. |
| 22 | | | 41 | 48 | 42 | 30.43 | 30.38 | 62 | 62 | W | WSW | Foggy | Cloud. | |
| 23 | ● | | 44 | 48 | 44 | 30.30 | 30.12 | 66 | 61 | WSW | SSW | Foggy | Cloud. | |
| 24 | | .10 | 45 | 49 | 44 | 29.94 | 29.94 | 68 | 70 | SW | W | Rain | | Sho'ry |
| 25 | | | 45 | 49 | 42 | 30.04 | 30.13 | 66 | 65 | NW | NNW | Cloudy | Fine | |
| 26 | | | 44 | 48 | 38 | 30.11 | 30.22 | 63 | 60 | NW | N | Fine | | Cloud. |
| 27 | | | 41 | 42 | 42 | 30.34 | 30.30 | 59 | 65 | N | W | VeryFog. | Cloud. | |
| 28 | | | 44 | 48 | 39 | 30.20 | 30.21 | 75 | 70 | NW | N | Foggy | Fine | |
| 29 | | | 38 | 48 | 36 | 30.21 | 30.27 | 67 | 60 | NW | N | Foggy | Cloud | Fine |
| 30 | ☾ | | 37 | 44 | 32 | 30.34 | 30.36 | 64 | 67 | NNE | WNW | Foggy | | |
| 31 | | .02 | 34 | 40 | 38 | 30.32 | 30.29 | 67 | 61 | W | W | Foggy | Fine | Cloud. |
| Feb. 1 | | | 41 | 45 | 40 | 30.18 | 30.05 | 62 | 62 | SW | WSW | Fine | | |
| 2 | | | 47 | 50 | 43 | 29.82 | 29.60 | 67 | 68 | WSW | W var. | Cloudy | | Hurrican. of W,&R. |
| 3 | | .30 | 47 | 48 | 36 | 29.51 | 29.68 | 66 | 61 | W var. | W | Fi.Windstr. | | |
| 4 | | .07 | 58 | 48 | 45 | 29.80 | 29.38 | 62 | 72 | W | SW | Foggy | Sho'ry | Rain |
| 5 | | | 48 | 50 | 35 | 29.31 | 29.85 | 64 | 58 | SW va. | W var. | Cloudy | Hur.Wi | Fine |
| 6 | ○ | .07 | 36 | 45 | 40 | 30.18 | 30.03 | 61 | 55 | W | S var. | Foggy | Fine | |
| 7 | | .10 | 43 | 50 | 46 | 29.90 | 29.83 | 63 | 70 | SSW | SW | Cloudy | Fine | Rain |
| 8 | | | 46 | 48 | 44 | 29.81 | 29.98 | 70 | 65 | WSW | SW | Showery | Fine | Cloud. |
| 9 | | | 45 | 52 | 45 | 29.92 | 29.89 | 67 | 64 | SW | SSW | Cloudy | | |
| 10 | | .07 | 47 | 51 | 44 | 29.87 | 29.82 | 67 | 65 | SSW | SSW | Cloudy | Fine | Sho'ry |
| 11 | | .15 | 46 | 51 | 40 | 29.98 | 30.15 | 65 | 65 | WSW | SW | Cloudy | Misty | Rain |
| 12 | | | 43 | 51 | 38 | 30.24 | 30.23 | 70 | 75 | WSW | E | VeryFog. | Foggy | Cloud. |
| 13 | | | 41 | 47 | 43 | 30.19 | 30.11 | 72 | 67 | SE | S | Cloudy | Fine | Cloud. |
| 14 | ☽ | | 45 | 51 | 42 | 30.11 | 30.10 | 63 | 71 | S | SW | Foggy | Cloud. | Fine |
| 15 | | .04 | 44 | 52 | 42 | 30.13 | 30.17 | 70 | 66 | SW | W | Fine | | Sho'ry |
| 16 | | | 44 | 51 | 47 | 30.35 | 30.34 | 67 | 66 | WNW | W | Foggy | Fine | Cloud. |
| 17 | | | 49 | 53 | 43 | 30.40 | 30.42 | 66 | 65 | NW | W | Fine | | |
| 18 | | .03 | 45 | 53 | 41 | 30.40 | 30.38 | 65 | 67 | SW | W | Fine | | |
| 19 | | | 43 | 51 | 42 | 30.41 | 30.30 | 57 | 61 | NNE | W | Fine | | |

The quantity of rain during the month of January, was 0.55 of an inch.

